# The function of chromatin modifiers in lineage commitment and cell fate specification

**DOI:** 10.1111/febs.13132

**Published:** 2014-11-20

**Authors:** Jason Signolet, Brian Hendrich

**Affiliations:** 1 Wellcome Trust – Medical Research Council Cambridge Stem Cell Institute, University of CambridgeUK; 2 Department of Biochemistry, University of CambridgeUK

**Keywords:** development, gene regulatory network, gene expression, NuRD, polycomb, stem cells

## Abstract

Proteins that modify the structure of chromatin are known to be important for various aspects of metazoan biology including development, disease and possibly ageing. Yet functional details of why these proteins are important, i.e. how their action influences a given biological process, are lacking. While it is now possible to describe the biochemistry of how these proteins remodel chromatin, their chromatin binding profiles in cell lines, or gene expression changes upon loss of a given protein, in very few cases has this easily translated into an understanding of how the function of that protein actually influences a developmental process. Given that many chromatin modifying proteins will largely exert their influence through control of gene expression, it is useful to consider developmental processes as changes in the gene regulatory network (GRN), with each cell type exhibiting a unique gene expression profile. In this essay we consider the impact of two abundant and highly conserved chromatin modifying complexes, namely the nucleosome remodelling and deacetylation (NuRD) complex and the polycomb repressive complex 2 (PRC2), on the change in GRNs associated with lineage commitment during early mammalian development. We propose that while the NuRD complex limits the stability of cell states and defines the developmental trajectory between two stable states, PRC2 activity is important for stabilizing a new GRN once established. Although these two complexes display different biochemical activities, chromatin binding profiles and mutant phenotypes, we propose a model to explain how they cooperate to facilitate the transition through cell states that is development.

## Introduction

Each multicellular organism arises from a single cell. During development, as cells divide and their numbers multiply, different groups of cells take on different roles. These different roles require cells to be able to respond to different signals, and often they will need to acquire drastically different morphologies to perform their roles adequately. Nearly all cells within an organism carry the same genome, yet each cell type has a distinct profile of gene expression to make it most fit for purpose: each cell type will have a distinct combination of genes which are on, off, primed or oscillating. Despite all of this heterogeneity and potential variability, animal development is normally very predictable. This suggests that the ability to transit between distinct gene expression profiles in a specific order is highly robust.

One of the most powerful available models to study differentiation between stably self-renewing cell types is the embryonic stem (ES) cell. ES cells are cells derived from the inner cell mass of a blastocyst-stage embryo [1]. They can self-renew indefinitely *in vitro*, and are prized because they promise the potential to form any type of somatic tissue (i.e. they are pluripotent). Indeed, the field of stem cell biology holds immense potential to positively impact human health through regenerative medicine [2–4], where stem cells could, in theory, be used to create any tissue type of need for the medical and/or pharmaceutical industry. However, in order to achieve this very broad aim, we must be able to both understand and control human development. In order to control development, we must be able to understand and control gene expression.

From observation, we know that an individual cell is able to react appropriately to signals from its surroundings whilst also being able to act upon internal programmes. Thus, it is clear that cells are capable of integrating multiple forms of information and are able to compute decisions based on this. We know that ordinary, wild-type ES cells are capable of self-renewal and differentiation. Furthermore, they have the capability to differentiate into more than one cell type, such that in certain culture conditions they can apparently choose between different fates, resulting in heterogeneous cultures. That is, cells which are exposed to seemingly identical conditions can exhibit different behaviours. So the question here is: how are these cells choosing one fate over another? Or, more precisely, by what mechanisms are the cells integrating and interpreting signals from their surroundings, and how can these interpretations result in different behaviours between cells in a clonal cell population?

One of the key mechanisms in allowing cells to respond to instructions and to modulate gene transcription is the chromatin modifying machinery. Complexes such as the nucleosome remodelling and deacetylation (NuRD) complex and the polycomb repressive complexes (PRC1/PRC2) are capable of remodelling and/or modifying chromatin, and all play important roles in cell differentiation. Previous reviews have focused on the physical changes to chromatin which accompany differentiation. In this review we consider what roles chromatin modifying complexes play in ES cell differentiation, but with a focus on the potential effects of these complexes on the dynamics of the transcription factor gene regulatory network (GRN) rather than on the physical chromatin itself.

## Mouse embryonic stem cells

Mouse ES cells have a relatively open chromatin structure which becomes denser upon differentiation. During differentiation, there is a wide-scale repression of ES-cell-state-related genes. This is achieved by a combination of factors, but the two we shall consider most closely here are histone modifications and nucleosome remodelling.

As well as allowing genome packaging, nucleosomes are employed in the regulation of transcription [5–7]. Nucleosome-level regulation is broadly concerned with controlling access of polymerases and transcription factors, increasing or decreasing the likelihood of transcription. This can be achieved by changing the location of nucleosomes with a nucleosome remodeller (e.g. by Chd4 in the NuRD complex or Brg1 in esBAF [5,8,9]) or by chemically modifying the histones at their N-terminal tails or globular domains (e.g. H3K27me3 by PRC2 [10,11]). All chromatin modifications like this are reversible and dynamic, although some can be maintained over many generations, giving rise to a type of cellular memory [12].

In standard culture conditions, and even in the relatively homogeneous, chemically defined two-inhibitor (2i) culture condition [13,14], mouseES cells are not completely homogeneous [15–18]. If we have a population of ES cells which spawn from a single cell, which self-renew, and which are morphologically indistinguishable from one another, we will nevertheless find subpopulations with discretely different gene expression profiles. Furthermore, these different patterns of expression can bias the cell towards self-renewal or differentiation, but at the same time the patterns can be dynamic and transient, creating temporal windows-of-opportunity for choosing a particular fate [19,20]. This already gives us some insight to the dynamics of the system.

The heterogeneity of gene expression seen in ES cells is often attributed to a combination of cellular noise, oscillating circuits [21] and various switches embedded in the GRN [19,22]. The concept of cellular noise intends to capture all of the factors which lead to random variability in cells – something of a formal acknowledgement of the messiness of biology [23–25]. Within this, sources of noise are often classed as intrinsic or extrinsic, although the boundary between the classes can be blurred [26–28].

In practice, factors which introduce intrinsic noise introduce variation to intracellular events which should be identically regulated (such as the random diffusion of macromolecules causing localized changes in the equilibria of biomolecular reactions [29]). Extrinsic noise refers to the variation of identically regulated events across a population of cells. Examples of this include differences in cell-cycle stage, differences in organelle distribution, or any environmental stimuli. This physical messiness gives a plausible driver for ES cell heterogeneity.

One of the most interesting aspects of ES cell heterogeneity is the fact that cells appear to be able to express many genes at broadly discrete levels (we very often find two steady states – to simplify, ON or OFF) and that, when measured across multiple generations, clonal cell populations can shift dynamically between the levels. The expression of the transcription factor NANOG is probably the most intensively studied example of this behaviour [16,17,30–32]. Multiple models have been proposed to explain how the dynamic switching of NANOG expression occurs, but as yet none fully captures the effect. Alongside NANOG, several other ‘pluripotency-related’ transcription factors are also heterogeneously expressed in mouseES cells, notably KLF4, KLF5 [33,34], REX1 (*Zfp42* [35,36]), TBX3 [34], ESRRB [1] and STELLA (*Dppa3*) [37].

## Cell types as high-dimensional attractors in gene expression state space

In a multicellular organism there can be hundreds of different identifiable cell types, and all of them will contain the same set of digitally encoded instructions: the genome. But what is a cell type? As a researcher, it is possible to class a cell based on its morphology, by the expression of certain genes, by the context in which it is found in, and by its behaviour in response to its environment. However, this does not address cell identity and the relationship between developmental lineages at a fundamental level [22,38].

**Figure 1:**
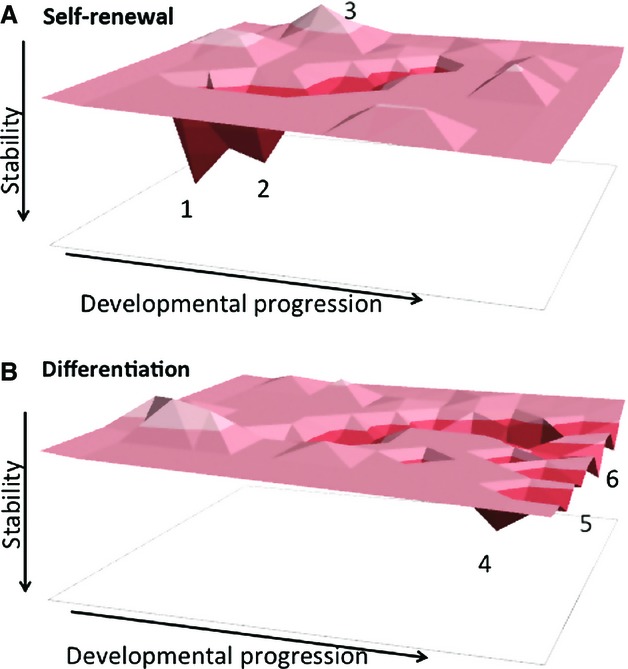
ES cell differentiation landscape. Model in which the GRN is indicated as a 3D surface, with all possible gene expression combinations existing as discrete coordinates in 2D state space. Some coordinates (meaning combinations of expression patterns) are more likely or more stable than others, and are called ‘attractors’. For example, in (A) positions 1 and 2 indicate stable or highly probable attractor states, whereas position 3 indicates a very unstable/unlikely position. Position 1 in (A) represents self-renewing cells in 2i/LIF conditions and position 2 represents ES cells in serum/LIF conditions. Upon loss of self-renewal signals (B), the resulting GRN no longer favours attractors 1 or 2, which become very unstable. In contrast attractors 4, 5 and 6 have become more stable and can attract cells traversing the landscape. These would represent entry points into different differentiation pathways. During normal development cells can only move from left to right in this model. Moving from right to left would only occur during experimental reprogramming. NuRD activity is predicted to limit the depth of the attractors and/or define the trajectories, displayed here as troughs, between attractors. PRC2 function is proposed to be required to stabilize/maintain the attractors.

Development is often described as if it is deterministic: zygote becomes morula becomes blastocyst etc. But, as alluded to above, there is a degree of stochasticity in development which is probably attributable to a combination of the often small numbers of each chemical species and biological noise [25,28,39]. This apparent paradox between developmental predictability and stochasticity can be resolved by considering the behaviour of a cell as a dynamical system [38,40,41]. One way of doing this based purely on gene expression is to categorize a cell's type by measuring its gene expression profile and assigning a cell state, *S*. The cell state is jointly defined by the expression of all genes in the genome *x*_1_, *x*_2_, …, *x*_*N*_, and so each state *S *= [*x*_1_, *x*_2_, …, *x*_*N*_] represents a coordinate in state space (Fig.1) [38]. Using this dynamical systems conception, different cell types thus occupy different regions in state space, and changes in expression are accompanied by the movement of *S* along one of a set of trajectories. GRNs include many nodes (genes) which directly influence the expression of other nodes, namely the transcription factors [42–44]. By nature this restricts the scope of potential trajectories.

We can consider that, due to gene expression noise, the expression of any gene *x*_*i*_ will fluctuate, and so the cell state *S* will move around in state space. If all genes were independent of each other, then *S* could be found at any coordinate. However, in biological systems, genes are not regulated totally independently of one another. In fact, there is a very high degree of inter-regulation, meaning that certain gene expression states are possible or even likely, whilst others are close to impossible to achieve. Thus, there are areas of state space which are more and less likely to be explored (Fig.1). Of the more likely areas of state space, there are points where all regulatory interactions are satisfied. These are stable points called attractors [38,40]. Once in an attractor, small perturbations in gene expression will not cause *x*_*i*_ to fluctuate too much, and so *S* is likely to fall back to the centre of the attractor. Large perturbations allow *S* to transit out of the basin of attraction, possibly towards another attractor. In this model, each different cell type would be an attractor in an ever evolving landscape of attractors [22].

The connections between nodes within the GRN can have a profound effect on the potential phenotypes of a cell and can influence the manner in which the cell can change phenotypes and differentiate. The attractor states encoded within the GRN topology are perhaps the most fundamental defining feature of cell type [24]. Next, we consider how chromatin modifying complexes can act upon the GRN, how they aid the transitions between cell types and how they allow the stabilization and establishment of differentiated cell types.

## Chromatin modifying complexes and the dynamics of the GRN

Here, we ask what role or roles a chromatin modifying complex occupies during the differentiation of cells, specifically ES cells. For the most part, the roles of chromatin modifying complexes have been described based on their physical effect on the chromatin. We can reduce differentiation and development to two central requirements: (a) the cell state must be able to transition from one stable state to another, with the differentiated state having some new ability (or set of abilities) required for further survival and/or growth; (b) the further transitions [i.e. further differentiation or the reverse transition (de-differentiation)] should normally be difficult to achieve, unless the correct environmental cues are present. We will refer to these two requirements as ‘transition’ and ‘establishment’.

We can think of differentiation as occurring when there is a bifurcation in phase space, i.e. something changes which causes a qualitative change in the behaviour of the system. Intuitively, chromatin remodellers could facilitate these bifurcation events in two broad ways. First, chromatin modifying complexes generally associate with a huge number of genetic control elements (promoters, enhancers etc.), and so their effect is felt at all points across the GRN. However, they also tend to have a high degree of specificity, and it is often observed (and assumed) that this specificity is mediated by complex transcription factor interactions. If this is so, chromatin modifiers should have a role in mediating the connections between nodes in the GRN. In doing this, the trajectories that a cell can take to move through phase space would be determined to some degree by chromatin modifying complexes. This could be seen as a form of intrinsic control over the transition stage of differentiation.

Second, genes within heterochromatic regions cannot be expressed. A gene in this condition has been effectively removed from the GRN, and the GRN has effectively been shrunk. In this establishment step, we would now expect the nature of the attractor states to change and for the reverse transition to be energetically unfavourable.

Considering cell states and developmental transition in this way allows us to predict phenotypes arising from various scenarios: if the transition is difficult, the cells will appear resistant to differentiation; if the establishment is difficult, the cells will be able to differentiate but appear to fail to commit to a new state; if too much of the required GRN is removed, then the cell may die or adopt a qualitatively wrong cell state; if too little of the GRN is turned off, then the cell may adopt a qualitatively wrong state.

We shall now explore how the mutant phenotypes of cells lacking two well-studied chromatin modifying complexes (NuRD and PRC2) would fit these descriptions and what possible further insights these would offer (for detailed reviews of the molecular biology of these complexes, see [5,9,10]).

## The NuRD complex

The NuRD complex is a multi-protein complex which is abundant in mammalian cells [45–47]. Its biochemically defined roles are to remodel chromatin and to deacetylate histones. Indeed, based on this histone deacetylase activity, it has commonly been referred to as a transcriptional co-repressor complex [45,47]. However, more recent evidence suggests that the presence of the complex at a gene locus is just as likely to be associated with active transcription as repressed transcription [48–51].

Mouse ES cells lacking the NuRD complex are, to an extent, stuck in the ‘ES’ cell state. That is, they self-renew but struggle to differentiate. Similarly, *in vivo*, NuRD-null epiblast cells fail to develop, resulting in failure to form the embryo proper. Whereas wild-type ES cells cultured in standard self-renewal conditions differentiate within two to three generations after withdrawal of leukaemia inhibitory factor (LIF), NuRD-deficient ES cells will continue to self-renew indefinitely [52]. Additionally, under standard ES cell culture conditions, wild-type-ES cell cultures consist of a heterogeneous mix of self-renewing and spontaneously differentiating cells, whereas NuRD-mutant ES cells do not show any signs of spontaneous differentiation. The NuRD complex also appears to have a high-level role in creating ES cell heterogeneity; one of the features of the NuRD-mutant ES cell phenotype is a loss of certain subpopulations as defined by transcription factor protein levels [34].

Where does NuRD function fit within our model of differentiation as a transition between stable states within the GRN? The NuRD complex (like all chromatin remodelling complexes) can be considered to be a general purpose tool which is employed by transcription factors at various gene loci to modify chromatin structure [53,54], acting globally to define the overall shape of the GRN. In this scenario, loss of the NuRD complex (or of any other chromatin remodelling complex) would alter the topology of the network. In the case of mouse pluripotent cells, absence of NuRD creates a more stable, more uniform ‘self-renewing’ state, where the probability that a cell can leave this state is much reduced. Thus NuRD can normally be considered to function to limit the stability of this pluripotent cell state, either by controlling the ‘depth’ of the ES cell state or by facilitating the transition away from the pluripotent cell state upon loss of self-renewal signals.

Could NuRD also be important for cells to maintain the identity of secondary cell states, i.e. those cells into which pluripotent cells differentiate? We find this to be less likely. One reason for this is that the NuRD-dependent differentiation seen in ES cells is context-dependent: under normal differentiation conditions, i.e. those to which pluripotent cells are exposed in an implantation-stage embryo, NuRD function is required for differentiation. In other contexts, such as upon treatment with differentiation-inducing drugs, injection under an adult kidney capsule or induction towards a trophectoderm fate, NuRD-deficient cells readily differentiate [52,55–57]. Further, while NuRD function is required for ES cells to form neural progenitor cells in culture, loss of NuRD in established neural progenitor cultures does not impair maintenance of these cells [52,58]. Therefore we favour a scenario in which control of gene expression by the NuRD complex is not important for maintaining the identity of somatic cell types. These examples also indicate that NuRD function is not absolutely required to define differentiation trajectories as, if given sufficiently strong extracellular signals, cells are able to find their way along a differentiation path. This is entirely consistent with NuRD defining the topology of the GRN, making the differentiation paths accessible to a pluripotent cell under normal, physiological conditions. However, if these cells are exposed to signals normally only seen much later in development (e.g. retinoic acid, or the extracellular milieu surrounding a kidney capsule), then this is sufficient to override any NuRD dependence upon differentiation.

Studies in somatic stem cell types support the notion that NuRD function defines the trajectories between cell states, but not that it influences the stability of a stem cell state. The founding NuRD component protein CHD4 (Mi-2β) has been shown to be important for developmental transitions in embryonic skin, i.e. for the normal progression of one epidermal progenitor cell to differentiate into another [59]. Similarly, deletion of *Chd4* in haematopoietic stem cells prevents neither self-renewal nor exiting the stem cell state; however, these stem cells produce an inappropriate mix of progenitor cell types [60]. Loss of the NuRD scaffold protein MBD3 during neural development results in a failure of neural progenitors to produce a normal complement of downstream cell types (Knock *et al*., in revision). Whilst it certainly is the case that the NuRD complex has tissue-specific behaviours and conformations [61–64], these studies are consistent with NuRD functioning to define differentiation trajectories in a number of different mammalian cell types.

## PRC2 and bivalent chromatin

PRC2 plays an essential role during embryonic development in mice and, indeed, in most other characterized metazoans (see for a recent review [10]). In mice, lack of the core PRC2 components EED, EZH2 or SUZ12 results in embryonic failure during the eighth or ninth day of development [65–67], placing its essential function slightly later than that of NuRD, which is required during the fifth day of development [55]. PRC2 maintains genes in a repressed state via the di- and tri-methylation of histone H3 at lysine 27 [68]. It has been noted that this mark, which is associated with transcriptional silencing, can coexist on promoters with a mark of actively transcribed genes, H3K4Me3, in a so-called ‘bivalent’ chromatin state [69–71]. Although this bivalent chromatin status was initially identified in mouseES cells and therefore thought to be a hallmark of stem cells, it was subsequently shown to also exist in differentiated cell types and is thus not a stem-cell-specific phenomenon [72,73].

The bivalent domain was first postulated to be a mark of promoters for which transcription was poised: not fully active (hence the H3K27Me3) and not fully repressed (due to the presence of H3K4Me3), but could quickly be turned either on or off depending upon which signals were received by the cell [71]. In this model of H3K27Me3 function, PRC2 could be seen to act to ensure cells remained responsive to differentiation signals, rather like the function described for NuRD above. Yet unlike NuRD, PRC2 components are not required for cells to exit the pluripotent state or for the onset of gastrulation during mouse embryogenesis [65–67]. Indeed, careful analyses of early epiblast cells in gastrulating embryos found that EED was not necessary for cells crossing the primitive streak to adopt their normal mesodermal fates, but defects were found in the function and behaviour of these resulting PRC2-mutant mesodermal cells [65,74]. These phenotypes do not appear consistent with a model in which PRC2, and by extension a bivalent chromatin state, is important for lineage fate choice.

Despite its general acceptance and popularity, a number of strands of evidence are beginning to cast doubt on the ‘bivalency-as-developmental poising’ model. ES cells lacking PRC2 components are able to self-renew without large-scale activation of differentiation-associated genes, demonstrating that PRC2 function (and, by extension, H3K27Me3) is not strictly required to prevent activation of this class of genes [75–78]. Culturing mouseES cells in conditions known to minimize transcriptional and functional heterogeneity, so-called 2i media [13,14], results in a reduction in global H3K27Me3 but no increase in precocious expression of lineage-specific transcription [79]. More compelling evidence came from deletion of the histone methyltransferase required for deposition of H3K4Me3 marks specifically at bivalent promoters, MLL2, which did not result in failure of developmental gene activation upon induction of ES cell differentiation [80]. This study provides strong genetic evidence that bivalency *per se* is not required for developmental priming of gene expression. A similar result was found when *Mll2* was knocked down in ES cells [81]. The importance of PRC2 function in early embryonic development is incontrovertible; however, the evidence does not support a role in specifying early cell fate decisions.

So how could the demonstrated function of PRC2 in mammalian development fit within our model of lineage commitment as a cell transiting between attractor states? PRC2 recruitment in ES cells is reliant on the transcriptional status of its target genes rather than by the action of any particular transcription factors [82]. It is associated with specific loci at different points in development, but this may be as a secondary effect to the genes being turned off by other means. As such, unlike NuRD, its primary function would be to maintain inactive genes in a silent, unresponsive state, and it would have less of an influence on the strength of connections within the network. This would serve to effectively remove nodes from the GRN and thus change the dynamics of the system. In other words, its primary role would be to maintain the stability (or ensure proper identity) of a specific cell state.

A function for PRC2 in stabilizing cell states is supported by the initial suggestion that lack of PRC2 components was incompatible with ES cell self-renewal [83]. It has subsequently become apparent that these ES cells actually are able to self-renew but show precocious differentiation and activation of some differentiation markers, i.e. cell state instability. Notably if these cells are cultured in 2i conditions they become considerably more stable [82]. That PRC2 functions in cell state stability is further supported by the behaviour of PRC2-mutant ES cells upon withdrawal of self-renewal signals. ES cells lacking the PRC2 component SUZ12 initially adopt gene expression profiles similar to their wild-type counterparts early in differentiation, consistent with them being able to both exit the self-renewing state and begin to transit to a new state (Fig.1B). However, after several days they revert to an ES-cell-like stable state [82]. This observation can be interpreted to indicate that without PRC2-mediated maintenance of transcriptional silencing the GRN is unable to properly stabilize in a non-ES-cell-like state.

## PRC2 and NuRD combine to define cell fates

Defining NuRD and PRC2 function in terms of how they influence the topology of the differentiation landscape, it is possible to make predictions about how the two would work together during differentiation. Specifically, NuRD activity would facilitate the exit of a cell from a given attractor state, either by destabilizing that attractor state or by defining differentiation trajectories, whereas PRC2 would ensure that once the cell arrives at a new state it would remain there.

One example of just such a partnership between NuRD and PRC2 is in the silencing of Hox loci in *Drosophila melanogaster*. During development, proteins encoded by the gap genes, such as Hunchback (Hb), maintain the appropriate spatial expression patterns of Hox gene expression by repressing their transcription outside of normal expression domains [84–86]. The transcriptional repressor Hb recruits the NuRD component protein dMi-2 to establish transcriptional silencing at Hox loci [87], thus establishing the positional identity of a cell. Although this expression pattern is maintained throughout development, expression of Hb is quickly lost after this establishment step and repression at these silenced Hox loci is maintained by PRC2. In the absence of PRC2 function the correct positional identity of cells is lost as appropriate Hox gene expression patterns are not maintained. Thus, in this case, NuRD and PRC2 act in sequential but separate steps of gene repression and cell fate determination: NuRD is used to establish the expression state, whereas PRC2 is used to maintain it.

A further example is provided in *Caenorhabditis elegans*, where orthologues of two NuRD components (Let418/Chd4 and Hda1/Hdac1) function to facilitate transition out of the germline state after fertilization [88]. That is, their activity is important to allow the genome to properly respond to differentiation signals which will instruct the fertilized egg to develop into an embryo, whereas maintenance of the specific cellular states is carried out, at least in part, by orthologues of the polycomb group proteins [88]. Remarkably, a similar function for NuRD component proteins had previously been described in *Arabidopsis thaliana*, in that a *Chd4* orthologue was shown to be important to prevent embryonic characteristics in somatic tissues [89–91].

How might such an order of events work mechanistically? One potential mechanism is provided by the seemingly complementary enzymatic activities of the two complexes. Transcriptional repression by NuRD is associated with loss of acetylation of H3K27, a mark of active transcription. This deacetylated H3K27 residue then becomes a substrate for the histone methyltransferase activity of PRC2, which facilitates mono-, di- and tri-methylation of H3K27 [92,93]. Therefore NuRD and PRC2 have the potential to act in tandem to change an active histone mark to a repressive mark. This mechanism was shown to occur at a subset of NuRD and PRC2 target genes in ES cells [94] and may well occur in somatic cells as well [95].

Genome-wide chromatin binding data in self-renewing mouseES cells show that NuRD components and PRC2 components colocalize at a relatively small subset of either complex's array of bound genomic locations [94]. This could mean that the two complexes do not tend to be present at the same loci at the same time, due to the sequential nature of their respective activities, or it could indicate that the examples described above represent a relatively infrequent occurrence of NuRD–PRC2 functional interaction. In the latter case we would predict that PRC2 and NuRD combine with other chromatin modifying protein complexes to carry out the functions described above, with the identities of these other complexes depending upon the cell type and lineage decision in question. Further, NuRD components are more likely to be found at actively transcribed genes than at silent genes [48–51]. This indicates that, most of the time, mammalian NuRD does not silence transcription as in the example of dMi2 and HOX genes in *Drosophila*, but rather modulates levels of active transcription [34,48]. Therefore it makes intuitive sense that only occasionally would this modulation require enforcement of transcriptional silencing by PRC2.

## Concluding remarks – and a get-out clause

Here we propose a hypothesis to explain how two chromatin modifying complexes influence the GRN and thereby function during mammalian development. This is based upon an existing model of ‘development as changes in the GRN’, which in turn is designed to help us better understand the biology we observe. We are aware that no model can fully describe every nuance of mammalian development nor take into consideration the >4500 citations in PubMed referring to NuRD and/or polycomb. The hypothesis we propose is designed to form a platform upon which to base further experiments and, like most good hypotheses, to be knocked down by clever experiments and replaced by another hypothesis based upon a more accurate model. It should also be pointed out that given the scale of genome-wide data on PRC2 and NuRD binding events and associated gene expression data, it will always be possible to find examples where these models and hypotheses do not hold true. Nevertheless we argue that these ideas do describe much of the phenotypic data present in the literature and are therefore useful.

The packaging of DNA into chromatin was a hugely important innovation in the evolution of eukaryotic organisms, and the regulation of chromatin has been shown time and again to be of crucial importance to cell survival and decision making. What has been less well explored is the role of chromatin repressive complexes in the regulation of the dynamics of the GRN. Admittedly, the dynamics of cell decision making is something of an open question, with suggestions and demonstrations of switching behaviours and oscillations at the local level, to critical-like self-organization at the level of the whole network [96,97]. What we have hoped to demonstrate is that, by stepping back from the molecular details, we may be able to understand the higher-level functions of these molecular machines and thereby better understand how the enzymatic activities contained within these complexes are harnessed to facilitate metazoan development.

There are a number of strands of thought that lead from this point. Much of the ability of a cell to compute decisions is presumed to be based on the dynamics of the system [98]. In a GRN, chromatin repressive complexes influence the expression of a large number of genes, placing them at the heart of cellular decision making. Therefore it is hoped that by concentrating on the higher-level effects of chromatin regulation in phase space we may gain entirely new insights into the use and evolution of chromatin-mediated regulation of cell fate decisions.

## Abbreviations

ES cell: embryonic stem cell
GRN: gene regulatory network
Hb: Hunchback
2i: two-inhibitor
LIF: leukocyte inhibitory factor
NuRD complex: nucleosome remodelling and deacetylation complex
PRC: polycomb repressive complex
